# Combining Predicted, Calculated, and Hands-On NMR Spectra to Enhance Instruction of Molecular Structure in Organic Chemistry

**DOI:** 10.1021/acs.jchemed.4c01565

**Published:** 2025-06-11

**Authors:** Larry Collins, Alexis R. Hartley, Christopher T. Jurgenson

**Affiliations:** † Department of Biological & Environmental Sciences, 3331 Longwood University, Farmville, Virginia 23909, United States; ‡ Division of Mathematics & Sciences, Delta State University, Cleveland, Mississippi 38733, United States

**Keywords:** NMR, molecular structure, organic chemistry, computational chemistry

## Abstract

NMR prediction using ChemDoodle, NMR calculation with Gaussian, and hands-on data collection using a benchtop NMR spectrometer were explored to assess their synergistic capacity in teaching NMR to organic chemistry students. Nine molecules representing functional groups commonly encountered in undergraduate organic chemistry were selected. Spectra obtained using each of the three methods were incorporated into a laboratory exercise where students utilized all methods to learn about NMR. Our approach combines experimental work with both an approximation and theoretical calculation providing students with a more comprehensive understanding. Ultimately, students learned how to perform NMR spectroscopy and how different computational tools can approach the problem in fundamentally different ways as outlined below. Our qualitative analysis reveals that students engaged in higher order thinking skills and reported several benefits to their learning.

## Graphical abstract

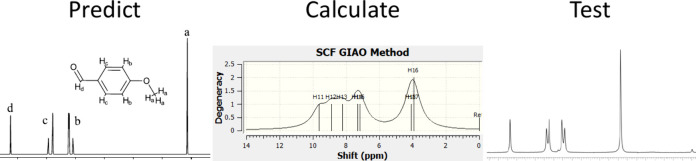

## Introduction

A core concept in every organic chemistry course is understanding molecular structure through NMR spectroscopy. For most students, this is the first example of a spectroscopic method that provides direct evidence of a molecule’s structure. While there is evidence that students can use NMR spectra to correctly interpret peaks in proton NMR spectra,^1^ it is still considered one of the most difficult topics of study in organic chemistry.^2^ Previous work on learning NMR has also highlighted how students approach the subject.^3^

Spatial ability and spatial intelligence are critical skills needed to take a series of peaks in an NMR spectrum and translate them into Kekulé or Lewis structures drawn on paper. Typically, students who study NMR use a spectrometer to collect spectra on molecules that are then interpreted based on rules associated with the noninteger spin nuclide’s environment (such as ^1^H and ^13^C). This takes into consideration molecular symmetry, degree of deshielding due to factors such as polarity and diamagnetic anisotropy, magnetic coupling, and peak integration.

Many textbook questions and interactive Web sites^4,5^ exist to hone the student’s ability to interpret NMR spectra, but even with these tools there remains a steep learning curve that many find difficult to overcome.^6^ Previous studies using eye-tracking software,^7,8^ analyzing instructor’s topic-specific pedagogical content knowledge,^2^ and using theories of scaffolding,^9^ interleaving, and blocking have attempted to pinpoint how students learn NMR. Our work builds on this literature by evaluating how hands-on NMR instruction can be enhanced by incorporating two additional techniques alongside it; NMR prediction using ChemDoodle and Gaussian for calculating spectra.

This study uses the 60Pro NMR from Nanalysis^10^ for the hands-on portion of the study. This is a 60 MHz NMR that runs on a standard 110 V outlet, has a built-in touchscreen, and utilizes a 1.41 T permanent magnet that operates at room temperature. It is capable of both ^1^H and ^13^C NMR. Many studies have been conducted using a benchtop NMR showing its robustness and utility,^11^ so for an academic laboratory this is an ideal solution that considers both practicality and cost. Current NMRs start at around $400 K for a 400 MHz spectrometer while the most basic ^1^H benchtop models can be purchased for less than $50K. That combined with the increasingly uncertain future of helium availability makes a benchtop NMR that requires no cryogens very attractive. However, there are major drawbacks that need to be addressed when using a benchtop NMR over a higher field NMR such as sensitivity and spin resolution due to having a weaker magnet. For the purposes of teaching NMR this is not a limitation for two reasons. 1) Much of the instrument’s use will be pedagogical allowing the instructor to choose samples that give interpretable spectra solely for teaching purposes. Any student or professor choosing to conduct research can still use a lower field NMR provided the sample does not have a highly complex structure, they allot ample time, and use a sample that can be concentrated enough to give a good signal. 2) Should the benchtop NMR be entirely incapable of generating interpretable spectra for a technically difficult sample, other options include accessing an NMR core facility at a large research university or using an NMR service company that does mail in analysis.

For the computational portion of this study, we used ChemDoodle to predict spectra and Gaussian to calculate NMR spectra. Both programs have a general user interface (GUI) for drawing molecules to generating spectra in silico. Any modern computer using Windows, MacOS, or Linux operating systems can run both suites of programs. One consideration is that ChemDoodle’s predictive NMR feature gives results quickly, while the NMR calculations using quantum mechanics in Gaussian takes much longer depending on the level of theory and computer hardware. However, an advantage of using Gaussian is that it uses quantum theory to predict chemical shifts without requiring a comprehensive understanding of the sophisticated underlying mathematics the program uses. The ease of use makes the running jobs with Gaussian teachable, which is why this software package was chosen for the project. Predicting NMR spectra can be done quickly since it relies on what is known from available ^1^H and ^13^C NMR spectra.^12–15^ The program suite ChemDoodle has a widget titled, “NMR Simulation” which allows the user to draw a molecule and see its associated predicted spectrum. Integration and J coupling is also automatically predicted. For more complicated structures the pulse frequency can be increased to gain better spin resolution. Use of the NMR Simulation feature can also serve as a control in the event the student makes a mistake during data collection. While convenient and fast, predicting spectra and measuring actual spectra do not give the exact same output. Predicted spectra that are significantly different from actual ones can indicate that a mistake was made, such as measuring the wrong sample, incorrect sample preparation, and unsuitable NMR data collection parameters, and thereby aid in troubleshooting problems when new users begin to learn how to operate an NMR.

Gaussian^16^ can also be used to generate NMR spectra using theoretical calculations. A molecule is built in GaussView^17^ then undergoes geometry optimization followed by NMR calculations using the Gaussian Calculation Setup feature. Different options such as the gauge invariant atomic orbital (GIAO), continuous set of gauge transformation (CSGT), and individual gages for atoms in molecules (IGAIM) methods may be selected.^18^ Level of theory and solvent model are also selected before the NMR spectra is calculated. Simple molecules may be calculated in a short period of time with a basic desktop or laptop computer. As with ChemDoodle, these spectra will not be precisely identical to the data collected by the benchtop NMR, but they serve to allow for comparisons since the relative chemical shifts and integrated proton values should be consistent between methods.

Our overall purpose for this integrated approach was to engage students in a hands-on laboratory experience with computational approaches to NMR. ChemDoodle approximates NMR spectra, while Gaussian uses quantum mechanical methods to calculate them. This approach combines the experimental work with both an approximation and a theoretical calculation where students can learn how to perform NMR spectroscopy, while also learning different computational tools that will approach the problem in fundamentally different ways as outlined above. The uses of ChemDoodle and Gaussian are both quite different in design and function, but using them in this combined approach enables students to learn how to use both. Thus, this provides students with an experience that gives them a comprehensive overview for understanding molecular structure through NMR spectroscopy.

## Materials and Methods

Several molecules were chosen to be representative of the types of functional groups that are encountered in an organic chemistry class shown in Table 1 and Figure 1. Each are available at high purity, low cost, and give simple spectra that are easily interpreted for students new to NMR.

**Table 1:** Example Molecule List

Functional group	Molecule	Source	Purity (%)
Hydrocarbon	Decane	Fisher	99.2
Haloalkene	1-Bromo-2-methylpropane	Aldrich	99
Ketone	Butanone	Fisher	99
Ester	*n*-Butyl acetate	Acros	99+
Alcohol	Isopentyl alcohol	Fisher	99
Aldehyde	p-Anisaldehyde	Acros	99+
Amide	*N*,*N*-Dimethylformamide	Aldrich	99.9+
Ether	*t*-Butylmethyl ether	Aldrich	99
Aromatic	Ethylbenzene	Fisher	″Certified″

**Figure 1:**
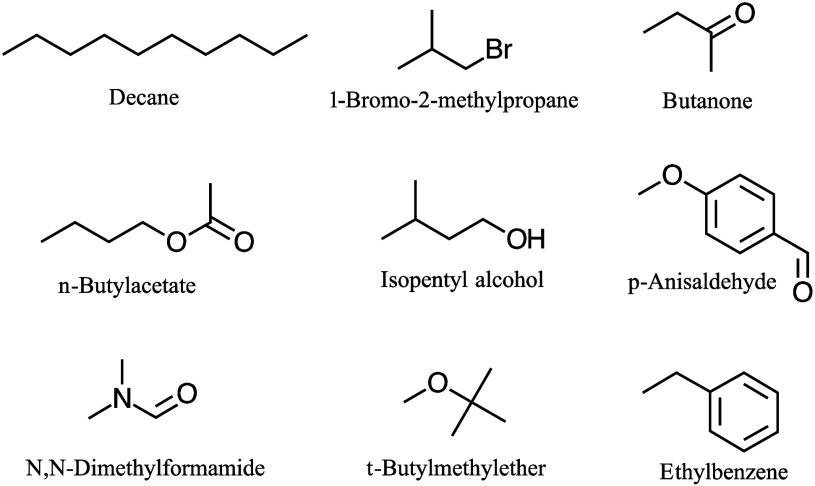
Example molecule structures.

NMR spectra predictions were done using the NMR Simulation which is a widget feature in ChemDoodle. After demonstrating how to use ChemDoodle, this assignment was done by the students as an independent exercise. The molecule is built in the GUI and transferred to the NMR simulation window where proton and Carbon-13 spectra are instantly predicted. It is possible to change parameters such as pulse frequency, temperature, and solvent, but for this study the default parameters for temperature and solvent were kept (temperature = 298 K, and solvent = CDCl_3_) while the pulse frequency was changed 60 MHz from the default 200 MHz to match the field of our NMR.

The NMR instrument used for collecting spectra is the benchtop NMR spectrometer 60Pro by Nanalysis.^10^ Students both prepared samples for proton NMR and operated the instrument themselves. Samples were prepared by weighing 35 mg of each sample into an NMR tube and adding 0.75 mL of deuterated chloroform. The initial spectrum shown on the spectrometer’s touchscreen was interpreted by the class as it was displayed at the end of the analysis. Carbon-13 NMR samples were prepared by the instructor the same way only using 100 mg of each sample to get a stronger signal due to the low Carbon-13 abundance in molecules. These spectra were collected outside of lab by the instructor due to the long data collection times. Table 2 outlines the data collection parameters for both proton and Carbon-13 NMR. Additional processing to calculate integration and for analyzing shielding values on both the proton and Carbon-13 NMR spectra was done using NMRFx Analyst.^19^

**Table 2:** Data Collection Parameters for NMR Spectra Collection

Nuclide	^1^H	^13^C
Sample size	35 mg	100 mg
Diluent - CDCl_3_	0.75 mL	0.75 mL
Scan delay	15 s	0 s
Number of points	4096	16384
Number of scans	4	1024
Decoupling mode	on/^13^C	on/^1^H
Data collection time	89 s	7067 s

NMR calculations were done using a 32-bit version of Gaussian 16 and GaussView 6 on a Dell Precision 3660 running Windows 11 Enterprise with 32 GB of RAM and an Intel i7–13700 processor. Molecules drawn in ChemDoodle were saved as.gjf files which can be read into GaussView 6. Each student had the opportunity to work at the terminal running Gaussian and input the data for at least one of the molecules. Due to the complexity of theoretical calculations, this process was guided by the instructor to ensure the input data was entered correctly. Each molecule then underwent a geometry optimization calculation using the Hartree–Fock method with a 3–21G basis set. Optimized structures were used for calculations with the GIAO method, B3LYP DFT functional, and a 6–311++G­(2d,p) basis set. The resulting spectra were saved as.png files displaying proton and Carbon-13 shieldings with tetramethylsilane calculated at the same level of theory as a reference.

### Student Surveys

To assess student experience with this intervention, we conducted a survey that was administered through the Qualtrics platform using the procedures described below. This study was approved as IRB-exempt by the university’s institutional review board prior to the beginning of the study.

A pilot survey was developed to measure student perceptions of learning (written by the research team). The survey contained Likert-scale and open-ended questions. The survey was not developed within the context of an existing theoretical framework but designed to be exploratory and capture student experiences to understand the benefits of this work for curriculum and instructional purposes. In the beginning, students were asked to rate their knowledge of the topic. After participating in the intervention described above, students were asked how much they learned and did the intervention enhance their understanding of the content. See for a copy of each survey, the NMR lab procedure, instructions on how to run Gaussian, and a video on using ChemDoodle. Responses to each survey were collected in the Fall 2024 semester.

Undergraduate students at a public four-year institution in the southeastern United States participated in this survey. Students completing this survey were enrolled in a sophomore level Organic Chemistry course (N = 15) taught by one of the authors on this paper. Survey data were deidentified prior to analysis of the data and students who did not complete each assessment requirement were removed from the data set.

Given the pilot nature of this study, we conducted a thematic analysis on the survey questions via open coding. First, the researchers read the texts to determine themes that emerged from each open question.^20^ During this step, the researchers read the student responses from each question and identified themes that emerged from the data. Next, the researchers coded each response according to the themes that were identified. This resulted in a list of themes along with quotes that supported their existence. These themes were then summarized across all survey questions to answer the research questions. The coding of these data resulted in a percent agreement greater than 80% which is considered acceptable in chemistry education research.^21,22^ When there was a disagreement, the authors discussed it as an avenue toward reaching consensus.

## Results

NMR Simulation in ChemDoodle predicts both ^1^H and ^13^C peaks for a molecule drawn in the GUI and is the fastest of the three methods. The user can interact with the molecule and spectrum for better viewing and clarity. For example, the user can change the scale on the X and Y axes, and mousing over a H or C atom will highlight the corresponding peaks in the associated predicted spectrum (Figure 2). One significant limitation in ^1^H spectra prediction is that a multiplet like the one highlighted in Figure 2 does not show the correct peak splitting. For example, the methine hydrogen highlighted in Figure 2B shows a septet, but the actual peak should be a septet of triplets as indicated by the peak information in 2D.

**Figure 2:**
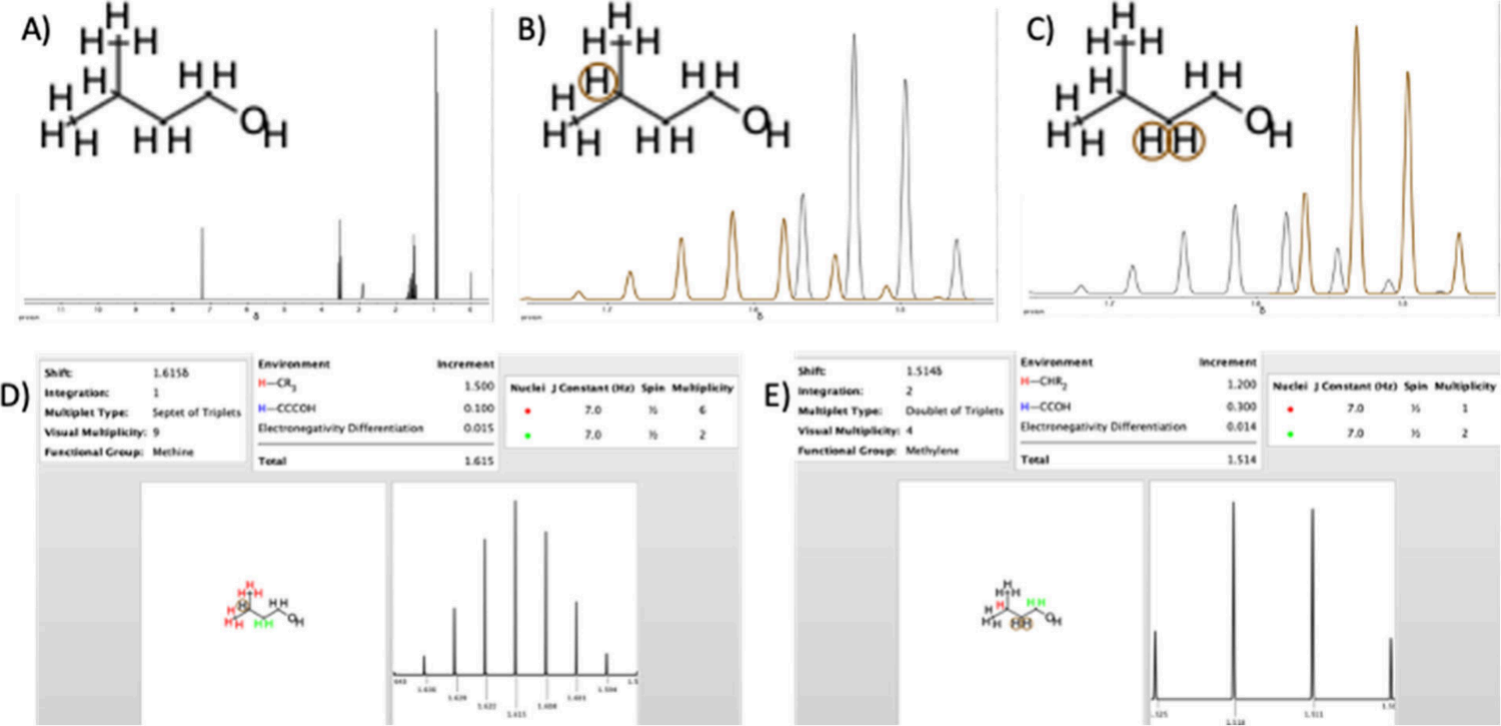
(A) Structure of isopentyl alcohol was drawn in the GUI then processed in the NMR Signal Seek window. The molecule with all hydrogens is shown with the predicted ^1^H spectra. A multiplet around 1.6δ is comprised of more than one peak that can be resolved by zooming in closer to this section of the spectrum. (B) Zooming in on the multiplet allows for highlighting which peaks correspond to which carbon atom by moving the cursor over the hydrogen atom in question. Here a sextet corresponding to the methine hydrogen atom is highlighted. (C) Moving the cursor over to the adjacent methylene hydrogen atoms highlights a predicted quartet. (D, E) A separate Peak Information window appears when clicking on each hydrogen atom that lists each chemical shift, integration, and multiplet type.

Spectra containing few hydrogen atoms typically exhibit well-resolved peaks (Figure 3). However, when numerous hydrogen atoms are present—especially those with similar chemical shifts, as is often the case in real NMR spectra—interpretation becomes more challenging. Multiplets can cause peaks to cluster closely together, making it difficult to distinguish individual hydrogen environments. Additionally, ChemDoodle does not display splitting patterns that follow the N + 1 rule, which omits valuable information about neighboring hydrogen atoms.

**Figure 3:**
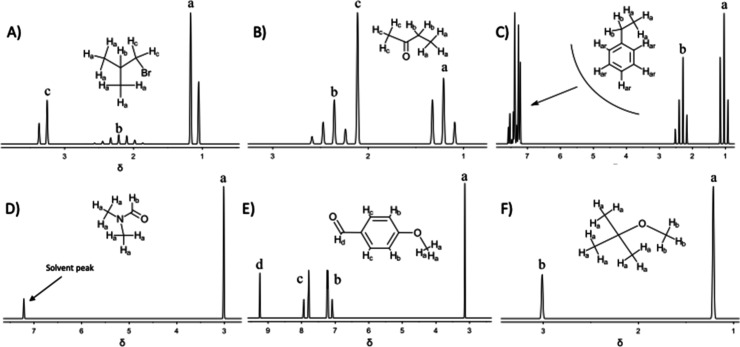
Predicted ^1^H spectra of (A) 1-bromo-2-methylpropane, (B) butanone, (C) ethylbenzene, (D) *N*,*N*-dimethylformamide [the peak at 7.2 ppm is not Hb, but rather is of the solvent], (E) p-anisaldehyde, and (F) *t*-butylmethyl ether.

Figure 4 highlights the predicted spectra of decane (4a), isopentyl alcohol (4b), and *n*-butyl acetate (4c). The advantage of the simulated spectra is that each individual peak can be highlighted along with their predicted chemical shift by using the feature discussed previously and shown in Figure 2.

**Figure 4:**
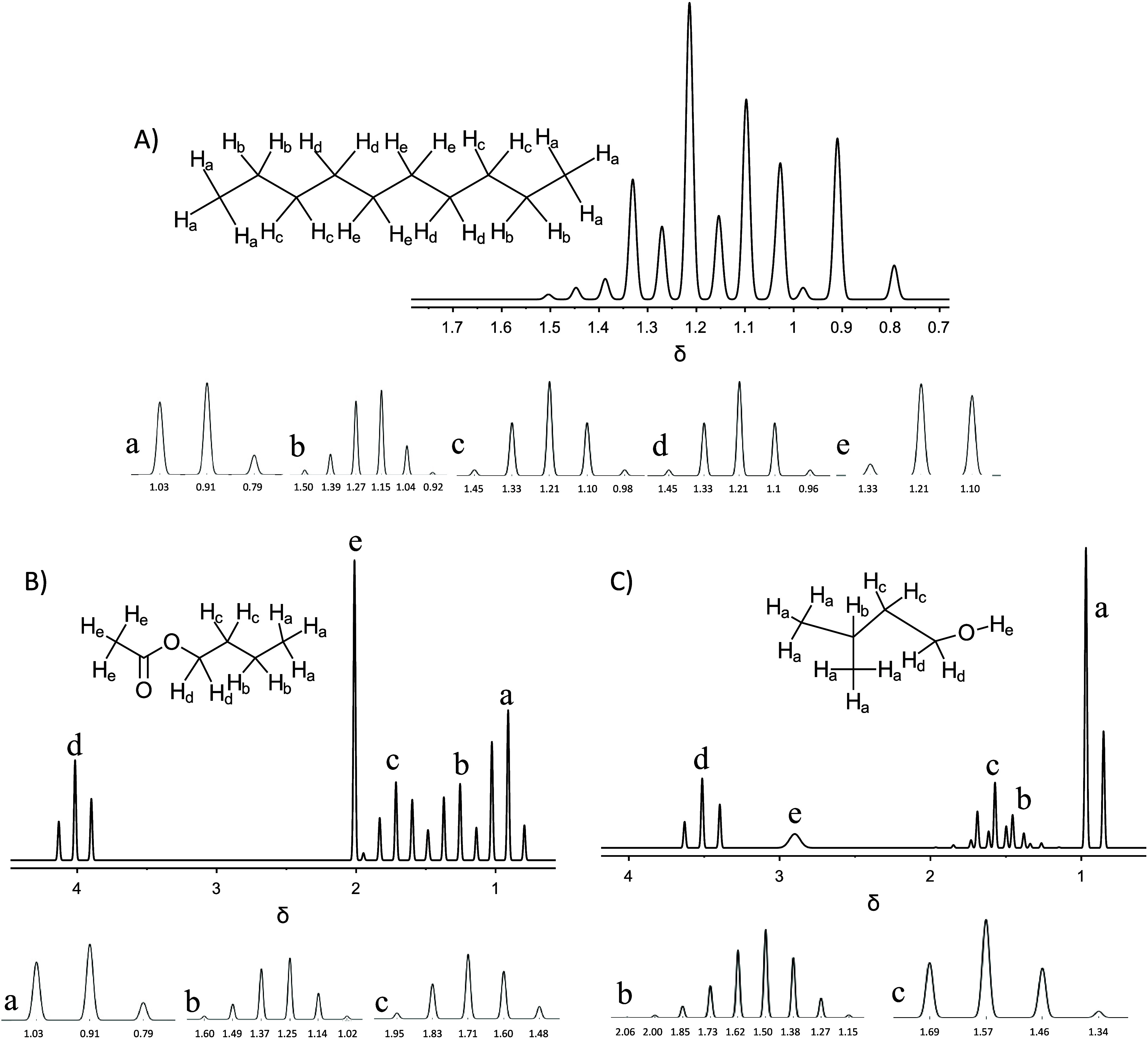
Predicted spectra of (A) decane, (B) *n*-butyl acetate and (C) isopentyl alcohol with individual peaks shown below each respective spectrum.

^13^C predictions are done simultaneously with ^1^H. The predicted ^13^C spectra are shown in Figure 5. There is a ^13^C triplet at 75–80 ppm that comes from splitting due to the deuterated chloroform solvent. This is what was used for sample preparation in the actual NMR data collection. Typically, NMR software removes these peaks, but they are present in the ChemDoodle spectra. This can be toggled off by choosing “no solvent” in the NMR widget settings which has no effect on the peaks in the resulting spectra.

**Figure 5:**
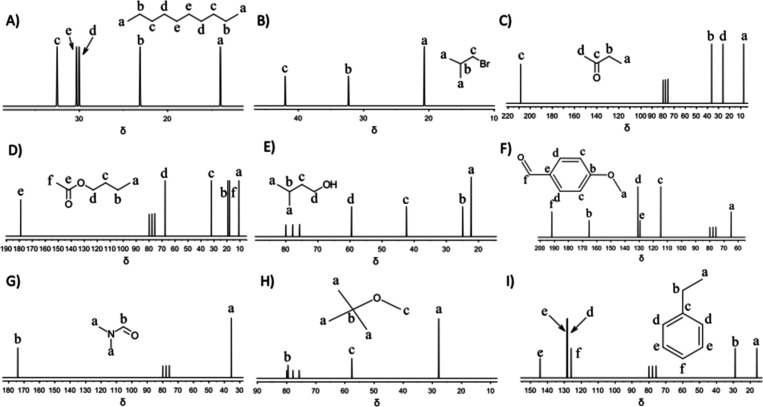
Predicted ^13^C spectra for (A) decane, (B) 1-bromo-2-methylpropane, (C) butanone, (D) *n*-butyl acetate, (E) isopentyl alcohol, (F) p-anisaldehyde, (G) *N*,*N*-dimethylformamide, (H) *t*-butylmethyl ether, and (I) ethylbenzene.

The NMR results for both the proton and Carbon-13 experiments are shown in Figures 6 and 7, respectively. The peak assignments are the same as shown with Figures 3–5. Integration for proton spectra is not automated, as each peak must be selected by clicking and dragging a window over each peak. The integrated value is highlighted above each selected peak.

**Figure 6:**
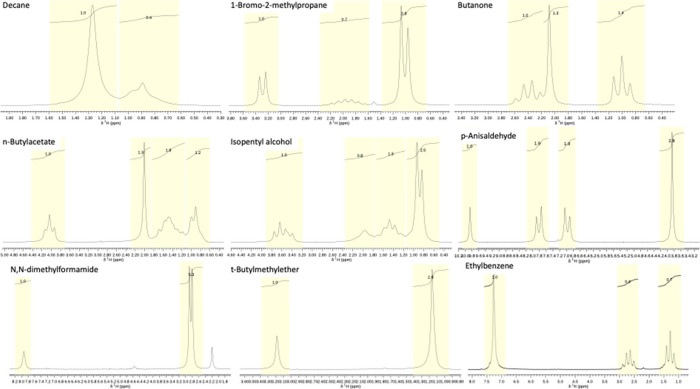
Proton NMR spectra

**Figure 7:**
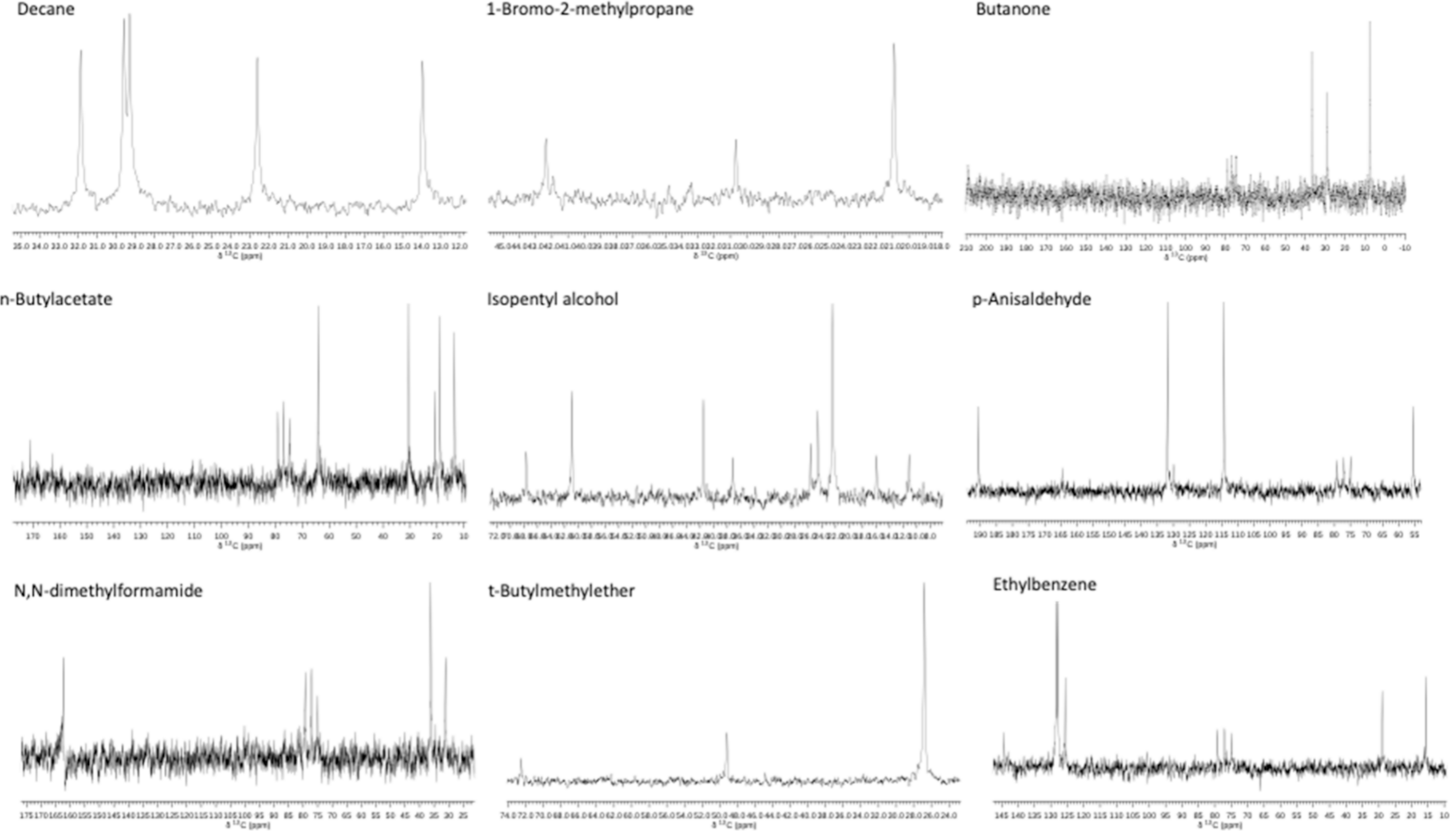
Carbon-13 spectra.

Carbon-13 peaks shown in Figure 7 match closely with standard values^5^ and show a triple peak from the deuterated chloroform around 75 ppm. The data collection time of approximately 2 h each makes this method unusable for more than one molecule in a typical undergraduate laboratory section. However, spectra can be taken at regular intervals over time since removing one sample, placing in a new one into the spectrometer, and beginning data collection only takes about a minute.

Calculated NMR spectra using Gaussian give very different looking spectra since the software is only capable of calculations on static structures. Because of this, chemical shifts of certain groups that would show up as a single peak have different values depending on the static structure environment of the geometry optimized conformation. For example, in 1-bromo-2-methylpropane the NMR shows three peaks – one peak from hydrogen atoms on the two methyl groups, one from hydrogen atoms on the methylene, and another from the hydrogen atom on methine (Figure 6). However, in the static calculated structure each hydrogen atom has a different shielding value resulting in a smooth calculated spectrum that lacks sharper peaks seen with dynamic NMR values (Figure 8A). This effect is especially pronounced in the methyl group closest to the bromine atom where the hydrogen shifts range from 2.00 on the hydrogen atom closest to bromine to 0.78 (Figure 8B).

**Figure 8:**
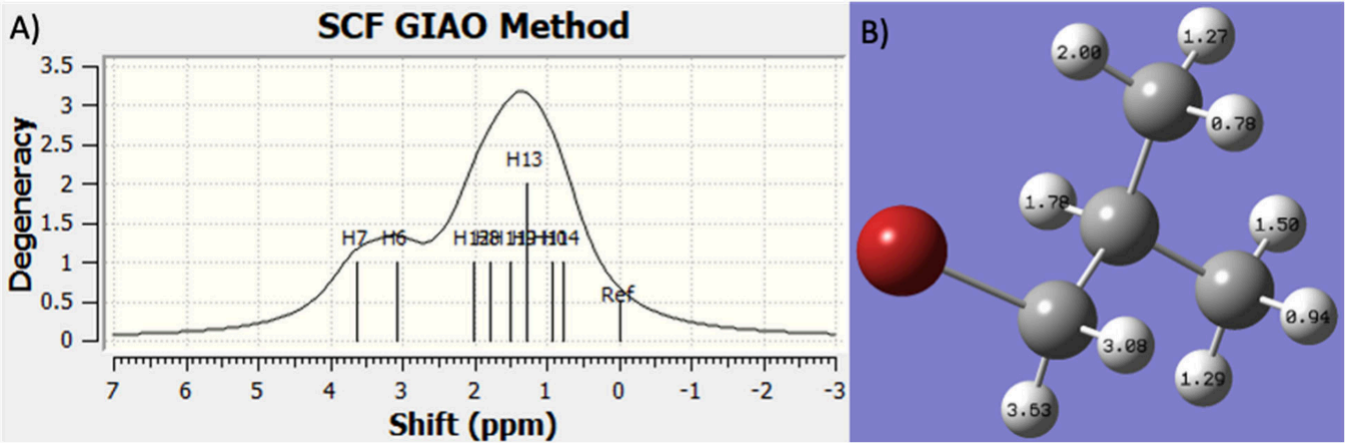
(A) Calculated NMR spectrum of 1-bromo-2-methylpropane. (B) Molecular structure showing the calculated shielding values on every hydrogen atom.

Because all the molecules with calculated proton shieldings are of static structures, hydrogen shieldings that would be equivalent in a dynamic structure were averaged and compared to the NMR and predictive methods shown in Table 3.

**Table 3:** NMR, Predicted, and Calculated Shielding Values for Each Molecule^b^

Molecule	Hydrogen	NMR	ChemDoodle	Gaussian
*t*-butylmethyl ether	Ha	1.16	1.21	1.37
	Hb	3.18	3.01	3.17
Butanone	Ha	1.00	1.21	1.60
	Hb	2.09	2.11	2.53
	Hc	2.41	2.41	2.33
Bromopropane	Ha	1.02	1.59	1.30
	Hb	1.97	1.64	1.78
	Hc	3.29	3.61	3.36
Ethylbenzene	Ha	1.28	1.04	0.84
	Hb	2.70	2.34	2.36
	Hc	7.26	7.38	7.31
p-anisaldehyde	Ha	3.86	3.14	4.00
	Hb	6.97	7.16	7.25
	Hc	7.82	7.87	8.56
	Hd	9.86	9.24	9.63
Decane	Ha	0.89	0.91	1.23
	Hb	1.27	1.21	1.39
	Hc	1.27	1.22	1.31
Isopentyl alcohol	Ha	0.89	0.91	1.11
	Hb	1.26	1.51	1.35
	Hc	1.48	1.61	1.50
	Hd	3.58	3.51	3.47
	He	1.96	2.90	1.63
DMF	Ha	2.84	3.01	2.72
	Hb	7.96	0^a^	8.64
*n*-butyl acetate	Ha	0.95	0.90	1.33
	Hb	1.49	1.31	1.36
	Hc	1.69	1.71	1.77
	Hd	4.04	4.02	3.85
	He	2.01	2.01	2.71

a Gaussian values are averaged for equivalent hydrogen atoms.

b The aldehydic hydrogen atom of DMF registers as a zero in ChemDoodle due to a lack of information in the NMR Simulation database.

In order to assess what the students learned, each was required to submit a laboratory report that included a purpose and procedure section describing the reason for conducting the experiment as well as the series of steps to be followed, a results section that included an interpretation of the data and observations from each method used to generate spectra and a conclusion summarizing what was learned. Students engaged with results of the spectral data from each method by identifying peaks based on chemical shift, splitting, and integration (for proton NMR only). Table 3 was given to them, and they were also to include a discussion of the differences between the three methods. All three methods varied in absolute chemical shift values for proton and Carbon-13, but the order of chemical shifts from least to most deshielded was preserved. However, there were two examples where the chemical shift values were swapped. This occurred where Gaussian swapped the order of Hb and Hc in both butanone and decane from the other two methods.

Each method offers distinct advantages and limitations. Hands-on NMR provides students with valuable experience in preparing real samples and operating an actual spectrometer. However, interpretation can be challenging due to the limited resolution of a 60 MHz instrument. ChemDoodle is user-friendly and generates interpreted spectra instantly, making it an excellent tool for quick analysis. Its main drawback is the absence of peak splitting according to the N + 1 rule, which is essential for understanding neighboring hydrogen environments. Gaussian, on the other hand, uses advanced quantum mechanical calculations to predict spectra. While highly sophisticated, it generates results based on static structures, leading to spectra that may differ significantly from those produced by experimental or approximated methods.

Students also completed a survey that captured how they perceived the innovation that we describe and its benefits to their learning. In this survey, we asked students to reflect on their experience by rating their knowledge of the topic and to explain how the innovation enhanced their understanding of the content and any other benefits that they identified by participating in this experience. Likert scale items revealed that 92% of students perceived using ChemDoodle and Gaussian as improving their learning in organic chemistry. 92% of students also identified that both approaches required them to think critically and supported them in meeting the learning outcomes as noted by their instructors. A further analysis of the qualitative data revealed that students did not necessarily prefer one approach over the other as 53% of students cited hands-on NMR as most beneficial to their learning, while 47% cited ChemDoodle and Gaussian as most beneficial. Qualitative responses did not further delineate any clear preference for ChemDoodle versus Gaussian. However, students cited that both approaches were beneficial to their learning as “multiple approaches made this easier to understand.” One student further explained that this combined approach permitted them to see and apply what they were discussing in lecture.

## Discussion

This intervention was designed for students in an upper-level organic chemistry class at a small four-year institution in the southeastern United States. Implementation involved 15 students completing the laboratory assignment, with 13 of them also completing the surveys—most of whom were college sophomores. To assess the effectiveness of this intervention, the survey that was conducted asked participants to evaluate their experiences and perceived effectiveness of this approach. There was no strong preference for one approach over another. However, our survey results indicate that students identified the importance of this combined approach to their overall understanding. This supports our rationale for using a combined approach to teach this concept. Furthermore, 100% of participants reported no perceived barriers to learning, while engaging in this intervention. These findings support the value that students found in this intervention.

In addition, participants reported positive gains in their metacognitive and spatial thinking skills in this survey. This is important as students see growth in their ability to monitor their own learning and understanding 3-dimensional chemistry phenomena. As one student noted, “being able to visualize the molecule and rotate them while also being able to highlight the peaks helped me check my understanding.” While the purpose of this paper is not to measure metacognitive or spatial thinking skills, it is worth noting that students perceived this as an additional gain by participating in the intervention. This supports the efficacy of this intervention beyond teaching chemistry content but also emphasizes other important scientific reasoning skills.

Input from students indicated that there was a variety of opinions on each method. No approach was unanimously liked or disliked. The students who favored the hands-on NMR appreciated conducting a real experiment and being able to interpret the results since NMR has real life applications. Students who favored ChemDoodle liked how simple it was to use and that it helped them learn how to draw structures of molecules correctly. An advantage of both Gaussian and ChemDoodle was that they are interactive and can display which hydrogen and carbon atoms are represented by peaks in the spectra. This helped students recognize where hydrogen atoms are, since many struggle with recognizing them using the bond-line representation commonly implemented in chemistry textbooks. Overall, students felt the variety of methods helped them better understand how to use NMR to learn about the structure of organic molecules. The static spectra given by Gaussian did not serve as an impediment to instruction since the outcome was discussed during the lab. One drawback to using Gaussian is the level of theory necessary to get interpretable spectra is outside the scope of an undergraduate course in organic chemistry. Because of this, setting up the calculation involves a very surface level discussion of what the program is doing. However, this is not a drawback simply because discussion of theory is not necessary to get interpretable spectra.

## Conclusions

The majority of students cited NMR as most beneficial to their learning. While the sample size is low (yet typical for exploratory qualitative work), the benefits toward student experience in organic chemistry were notable. Integrating this approach into an organic chemistry class provided students with an opportunity to visualize chemical phenomena and support their understanding of a challenging course concept. Students also made gains in their spatial thinking and metacognitive skills which further supports the efficacy of NMR in the classroom. In the future, using example molecules that have large differences in chemical shifts would make interpretation of spectra easier. When first learning NMR, using examples where each hydrogen atom produces a distinct and easily identifiable signal can make interpretation much more approachable. In contrast, compounds like decane—where many hydrogen and carbon atoms have similar chemical shifts—produce spectra with closely clustered peaks. This can be confusing for students who are just beginning to study NMR. Other molecules such as p-anisaldehyde and ethylbenzene have clear peaks separated by large differences in chemical shift, making discussion about the spectrum and molecular structure more straightforward. Another concern is that carbon-13 NMR takes so long it cannot be done during a typical undergraduate laboratory session for more than one or two molecules. Having a spectrometer capable of only proton NMR would not be an impediment to learning, since students would still get the hands-on experience with data collection and interpretation, as well as being a more affordable option.

## Supplementary Material











## Notes

NMR laboratory instructions (PDF, DOCX)
Instructions on how to use Gaussian to calculate NMR spectra (PDF, DOCX)
Video tutorial on using ChemDoodle to predict NMR spectra (MP4)
