# Supplementary material for: Combining Predicted, Calculated, and Hands-On NMR Spectra to Enhance Instruction of Molecular Structure in Organic Chemistry
Source: J Chem Educ. 2025 Jun 11;102(7):2777–85. doi: 10.1021/acs.jchemed.4c01565 (PMC12243081; doi:10.1021/acs.jchemed.4c01565)
Supplement: Supplementary file 1 [file ed4c01565_si_001.pdf]

Combining Predicted, Calculated, and Hands-On NMR Spectra to Enhance Instruction of Molecular Structure in Organic Chemistry

Larry Collins[1*], Alexis R. Hartley[2], and Christopher T. Jurgenson[2*]

1) Department of Biological & Environmental Sciences, Longwood University, Farmville VA 23909, United States; collinslb@longwood.edu
2) Division of Mathematics & Sciences, Delta State University, Cleveland MS 38733, United States; cjurgenson@deltastate.edu

*Corresponding authors

NMR Laboratory

Description:

We will be using NMR to collect spectra on several organic compounds. Each spectrum will be used to identify hydrogen and carbon atoms in the molecule.

Materials:

- 9 NMR tubes
- Deuterated chloroform
- 60pro benchtop NMR

Chemicals:

Functional group	Molecule	Source	Purity (%)
Hydrocarbon	Decane	Fisher	99.2
Haloalkene	1-Bromo-2-methylpropane	Aldrich	99
Ketone	Butanone	Fisher	99
Ester	n-Butylacetate	Acros	99+
Alcohol	Isopentyl alcohol	Fisher	99
Aldehyde	p-Anisaldehyde	Acros	99+
Amide	N,N-Dimethylformamide	Aldrich	99.9+
Ether	t-Butylmethylether	Aldrich	99
Aromatic	Ethylbenzene	Fisher	"Certified"

Proton NMR Method:

1. Weigh 35 mg of each sample into an NMR tube.
2. Add 0.75 mL of deuterated chloroform using a pipette.

Students will prepare the samples themselves so each group does at least one measurement.

Carbon-13 Method:

1. Samples are prepared the same way only using 100 mg of each sample to get a stronger signal due to the low Carbon-13 abundance in molecules.

These samples take much longer, so one sample will be prepared and shown to the class. The remaining spectra will be distributed to all students through the NMR module on Canvas.

The table below outlines the data collection parameters for both proton and Carbon-13 NMR.

Nuclide	^{1}H	^{13}C
Sample size	35 mg	100 mg
Diluent - $CDCl_3$	0.75 mL	0.75 mL
Scan delay	15 s	0 s
Number of points	4096	16384
Number of scans	4	1024

Decoupling mode	on/^{13}C	on/^{1}H
Data collection time	89 s	7067 s

Data processing:

The spectra generated from raw data will be integrated by Dr. Jurgenson and distributed to everyone through the NMR module on Canvas.

The lab report must include:

- Purpose and procedure
- Materials and methods
- All collected spectra from proton and carbon-13 NMR. You must identify every peak in each spectrum.
- Conclusion
