# Supplementary material for: Combining Predicted, Calculated, and Hands-On NMR Spectra to Enhance Instruction of Molecular Structure in Organic Chemistry
Source: J Chem Educ. 2025 Jun 11;102(7):2777–85. doi: 10.1021/acs.jchemed.4c01565 (PMC12243081; doi:10.1021/acs.jchemed.4c01565)

Combining Predicted, Calculated, and Hands-On NMR Spectra to Enhance Instruction of Molecular Structure in Organic Chemistry

Larry Collins[1*], Alexis R. Hartley[2], and Christopher T. Jurgenson[2*]

1) Department of Biological & Environmental Sciences, Longwood University, Farmville VA 23909, United States; collinslb@longwood.edu
2) Division of Mathematics & Sciences, Delta State University, Cleveland MS 38733, United States; cjurgenson@deltastate.edu

*Corresponding authors

Gaussian Instructions for Calculating NMR Spectra

- Load the .mol file for your molecule generated in ChemDoodle. Here we are using p-anisaldehyde.

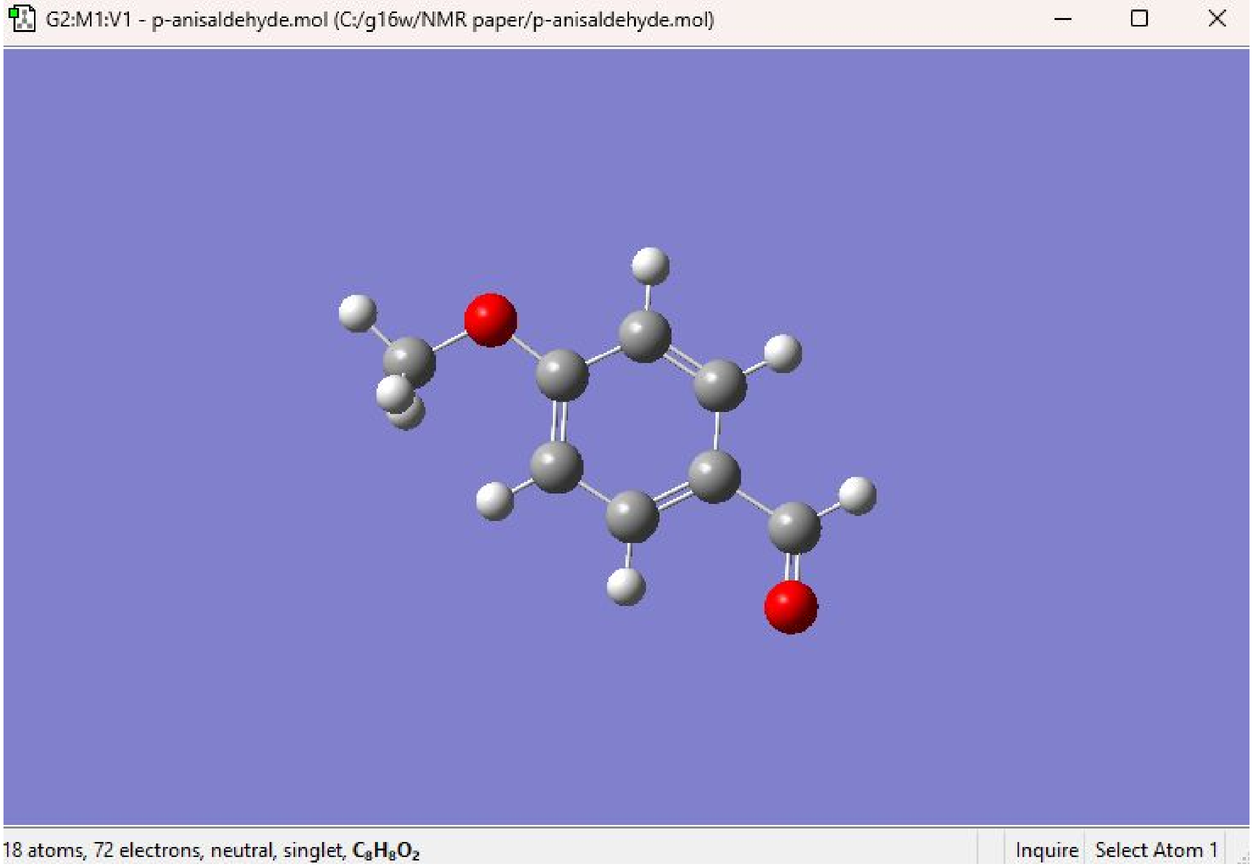

- Right click in the window and select Calculate >Gaussian Calculation Setup

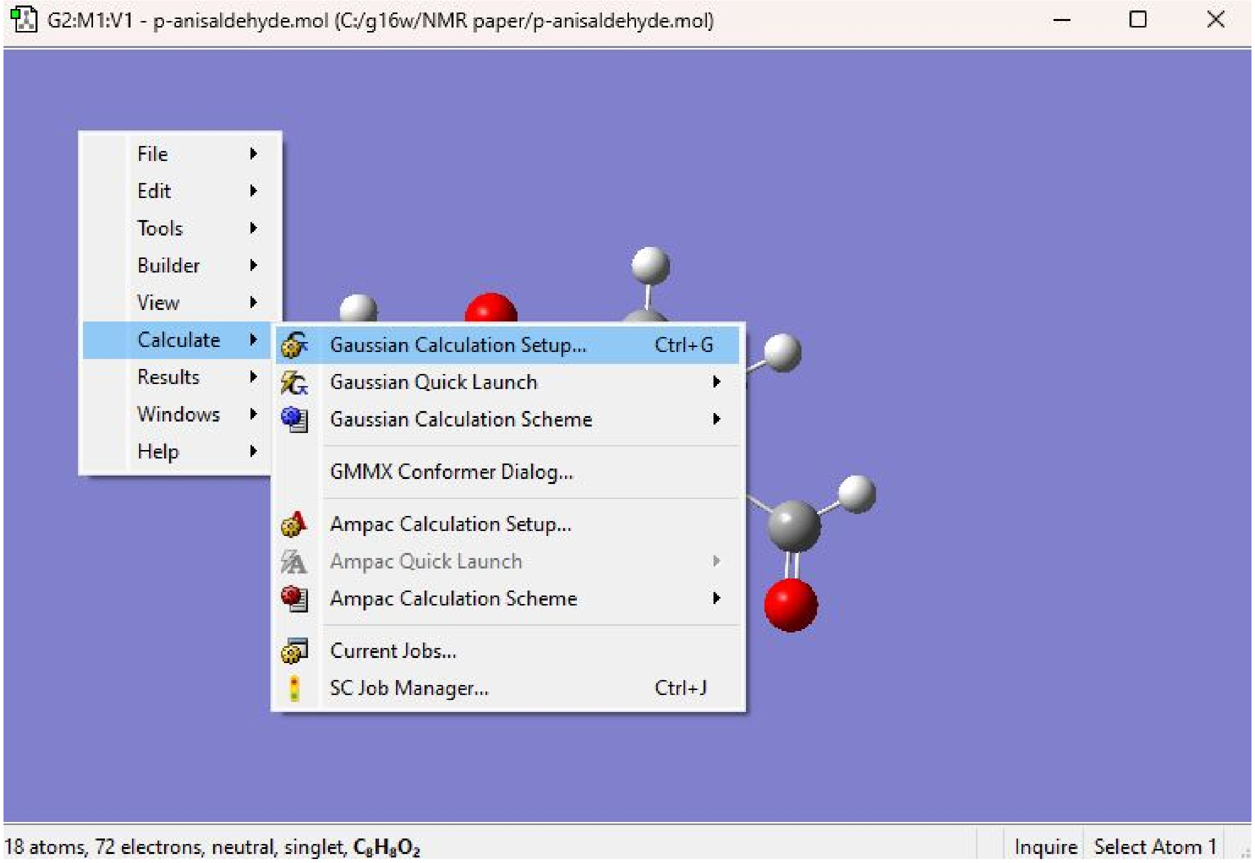

- Set the following parameters in the Gaussian Calculation Setup window:

- Job type > NMR

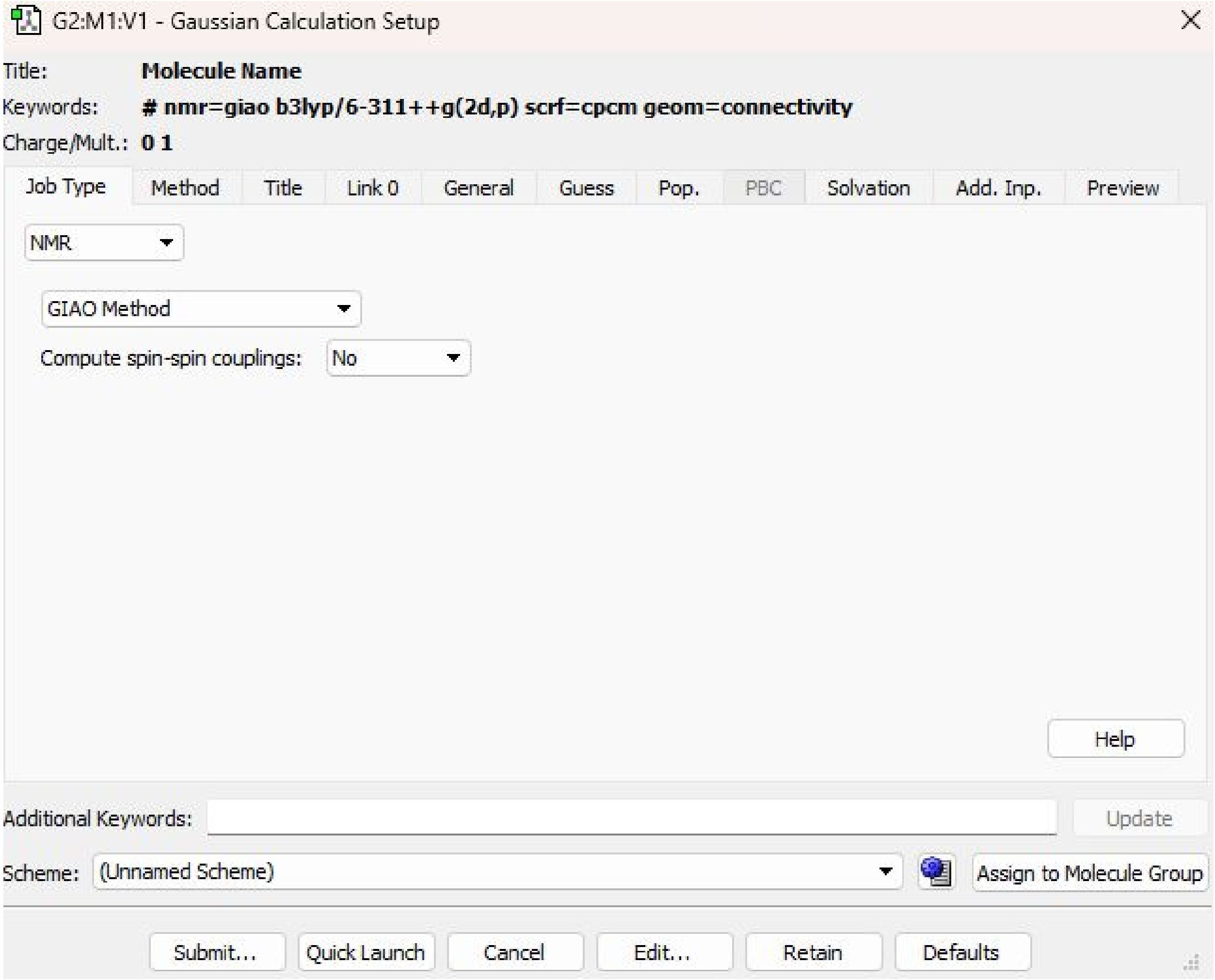
G2:M1:V1 - Gaussian Calculation Setup
Title: Molecule Name
Keywords: # nmr=giao b3lyp/6-311++g(2d,p) scrf=cpcm geom=connectivity
Charge/Mult.: 0 1
Job Type
Method
Title
Link 0
General
Guess
Pop.
PBC
Solvation
Add. Inp.
Preview
NMR
GIAO Method
Compute spin-spin couplings:
No
Help
Additional Keywords:
Update
Scheme:
(Unnamed Scheme)
Assign to Molecule Group
Submit...
Quick Launch
Cancel
Edit...
Retain
Defaults

- Method > Ground State, DFT, B3LYP, Basis Set 6-311G ++ 2d,p

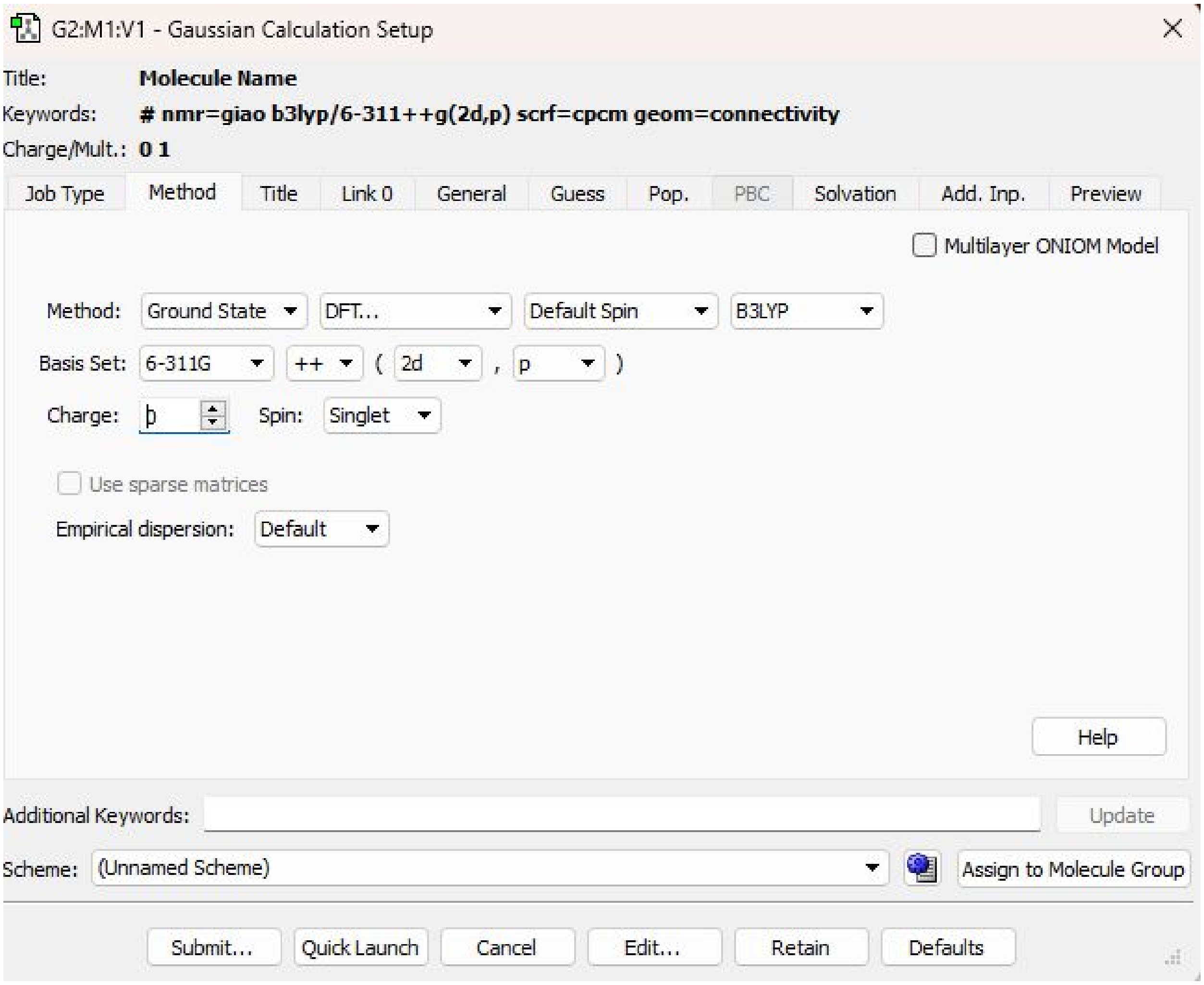

- Solvation > Model: CPCM

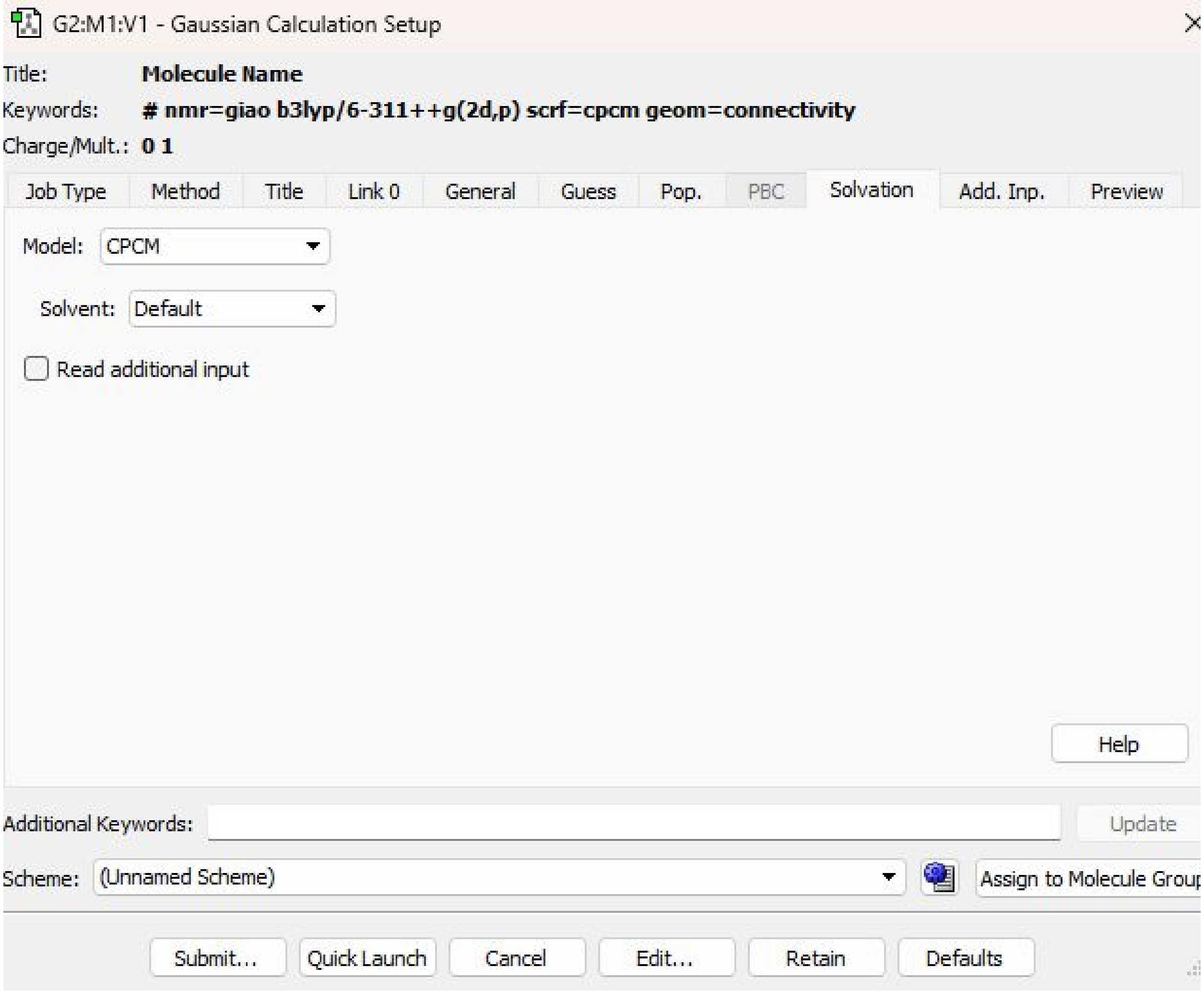

- Preview > Submit…

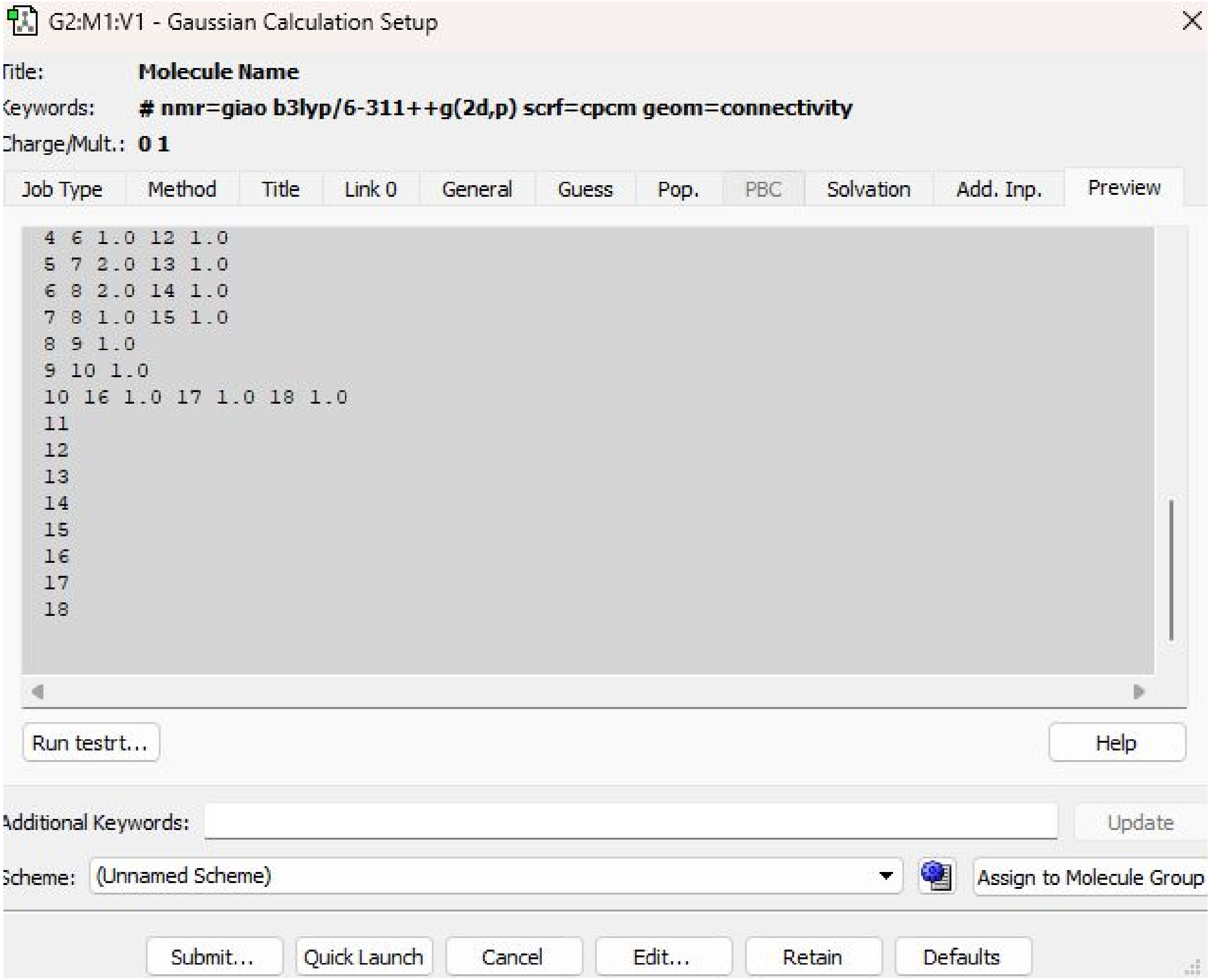

Supplement: Supplementary file 3 [file ed4c01565_si_003.pdf]
