# Supplementary material for: Combining Predicted, Calculated, and Hands-On NMR Spectra to Enhance Instruction of Molecular Structure in Organic Chemistry
Source: J Chem Educ. 2025 Jun 11;102(7):2777–85. doi: 10.1021/acs.jchemed.4c01565 (PMC12243081; doi:10.1021/acs.jchemed.4c01565)

Combining Predicted, Calculated, and Hands-On NMR Spectra to Enhance Instruction of Molecular Structure in Organic Chemistry

Larry Collins^1*^, Alexis R. Hartley^2^, and Christopher T. Jurgenson^2*^

1. Department of Biological & Environmental Sciences, Longwood University, Farmville VA 23909, United States; [collinslb@longwood.edu](mailto:collinslb@longwood.edu)
2. Division of Mathematics & Sciences, Delta State University, Cleveland MS 38733, United States; [cjurgenson@deltastate.edu](mailto:cjurgenson@deltastate.edu)

*Corresponding authors

Gaussian Instructions for Calculating NMR Spectra

- Load the .mol file for your molecule generated in ChemDoodle. Here we are using p-anisaldehyde.
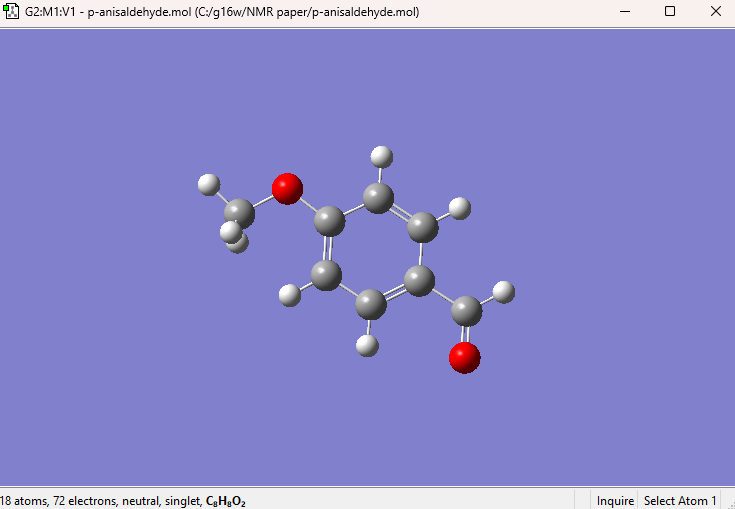

- Right click in the window and select Calculate >Gaussian Calculation Setup
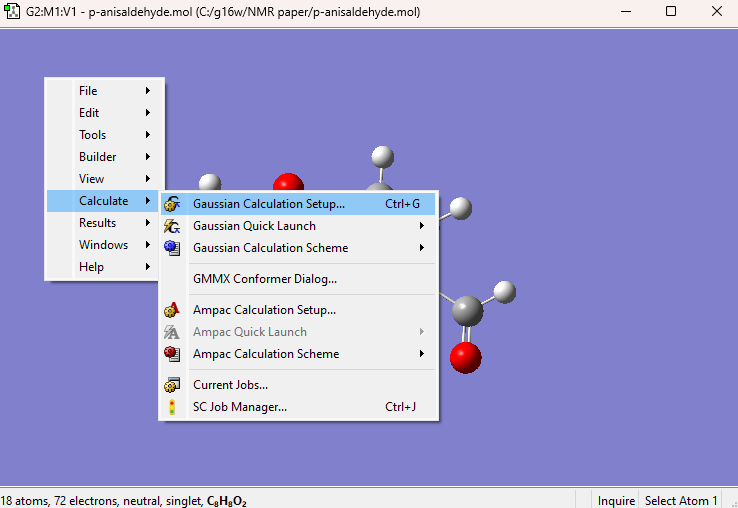

- Set the following parameters in the Gaussian Calculation Setup window:
  - Job type > NMR


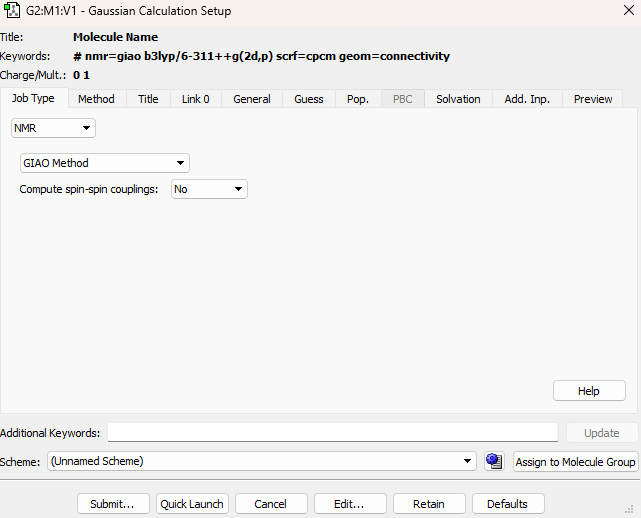


- -
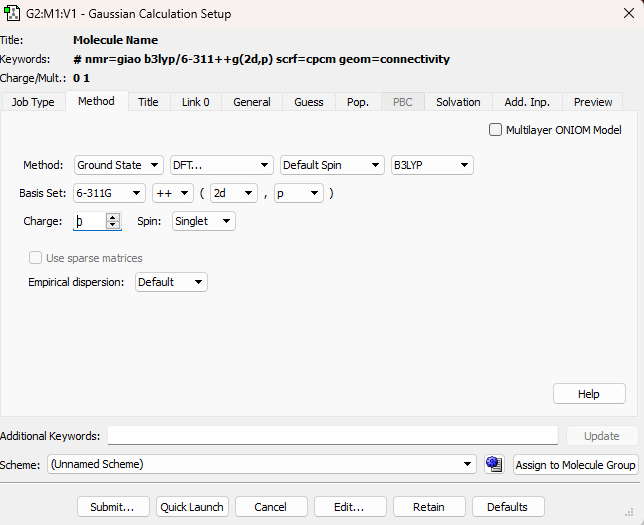
Method > Ground State, DFT, B3LYP, Basis Set 6-311G ++ 2d,p
  - Solvation > Model: CPCM


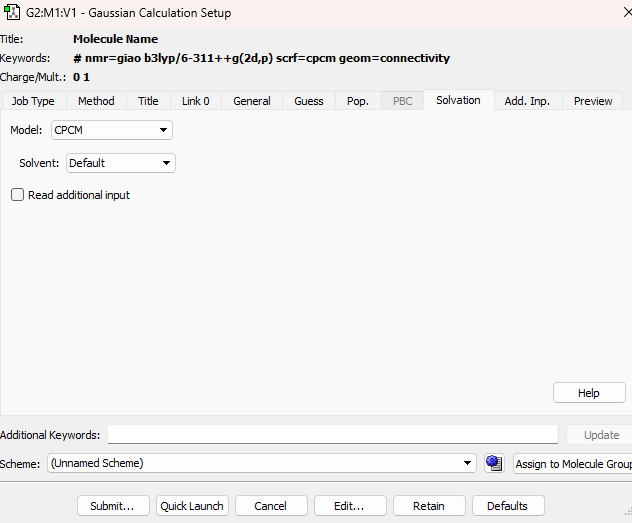


- - Preview > Submit…


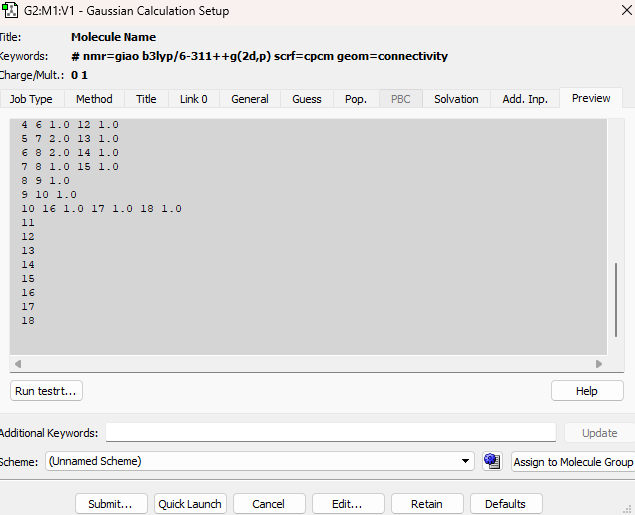

Supplement: Supplementary file 4 [file ed4c01565_si_004.docx]
